# Antireflective Coatings: Conventional Stacking Layers and Ultrathin Plasmonic Metasurfaces, A Mini-Review

**DOI:** 10.3390/ma9060497

**Published:** 2016-06-21

**Authors:** Mehdi Keshavarz Hedayati, Mady Elbahri

**Affiliations:** 1 Nanochemistry and Nanoengineering, Institute for Materials Science, Faculty of Engineering, Christian-Albrechts-Universität zu Kiel, Kiel 24143, Germany; 2 Nanochemistry and Nanoengineering, Helmholtz-Zentrum Geesthacht, Geesthacht 21502, Germany; 3 Nanochemistry and Nanoengineering, School of Chemical Technology, Aalto University, Kemistintie 1, Aalto 00076, Finland

**Keywords:** antireflective coating, plasmonic metasurface, absorbing antireflective coating, antireflection

## Abstract

Reduction of unwanted light reflection from a surface of a substance is very essential for improvement of the performance of optical and photonic devices. Antireflective coatings (ARCs) made of single or stacking layers of dielectrics, nano/microstructures or a mixture of both are the conventional design geometry for suppression of reflection. Recent progress in theoretical nanophotonics and nanofabrication has enabled more flexibility in design and fabrication of miniaturized coatings which has in turn advanced the field of ARCs considerably. In particular, the emergence of plasmonic and metasurfaces allows for the realization of broadband and angular-insensitive ARC coatings at an order of magnitude thinner than the operational wavelengths. In this review, a short overview of the development of ARCs, with particular attention paid to the state-of-the-art plasmonic- and metasurface-based antireflective surfaces, is presented.

## 1. Introduction

Refection of light (light-bending power) off the boundary (or at the interface) of two mediums is a desired phenomenon in mirrors but it is unfavorable in telescopes [1], lenses, and glasses [2] where it is considered as optical loss (absorption). For the latter cases, there has been an ongoing effort to reduce reflection by using some coating and patterns on the reflective surface, which is known as antireflective (or antireflection) coating (ARC). The development of ARCs began with Rayleigh in 1879, when he proposed that the reflectivity off the outer surface of an object could be reduced on the condition that the transition of refractive index (RI) between the object and surrounding medium is small [3]. Following the proposal of Rayleigh, English lens designer H. Dennis Taylor in 1892 saw that tarnished camera lenses permit photography with less exposure [4]. He discovered a decline in reflection linked to the coating (tarnish) on a glass surface [5]. This observation turned into a method of ARC development when F. E. Wright intentionally tarnished glass via chemical approaches. He described the mechanism of the anti-glare properties of the coating in terms of gradual RI transition from glass to air due to tarnishing [5].

Throughout the last century, various methods and strategies have been developed in order to lessen the reflection including (but not limited to) single-, double- or multiple layer ARCs as well as structuring/patterning of the surface. All of these methods are referred to throughout this review as conventional approaches as the principle and progress of each method is discussed.

The rapid development of modern optoelectronic devices such as displays, touchscreens and super-compact cameras, among others, place new demands on the ARC industry. The optic market requires three properties in particular of the ARCs: (1) ultrathin thickness; (2) insensitivity to the angle of incidence (non-iridescent); and (3) broadband. These requirements have recently been met by the emergence of the plasmonic and metasurface concept which is discussed in the present review.

## 2. Conventional Antireflective Coatings (ARCs)

### 2.1. Single-Layer ARC

In principle, one can calculate the intensity of the light which reflects off any surface (interface of any medium with surrounding environment) with the Fresnel equation [6]. Accordingly, a non-absorbing substance with refractive index (RI) equals *n**_s_*** at a desired wavelength; the RI and the thickness of the coating (also known as quarter-wavelength ARC) should fulfill the following conditions in order to reduce the reflection of the substrate down to zero at that wavelength:

(1) $$n_{arc}=\sqrt{n_{s}.n_{env}}$$

(2) $$d_{arc}=\frac{\lambda }{4\times n_{arc}}$$

in which *n**_env_*** and *n**_arc_*** are the RI of the surrounding medium and ARC and *d**_arc_*** is the thickness of the ARC, respectively. Note that the above equations are only valid for a homogeneous and non-absorbing medium; otherwise, the calculation gets more complicated due to the loss at each medium. For silicon (as the main absorbing material in the photovoltaic industry), in the middle of the visible spectrum 550 nm, one needs to deposit a 68 nm coating with an RI of 2.02 (e.g., *Si*_3_*N*_4_ [7]). Such a coating provides the reflection dip at 550 nm, whereas the average reflection through the visible spectrum would be 5.3%.

The major problem of single-layer quarter-wavelength ARCs is that they reduce the reflectivity for limited (almost single) wavelengths and incidence angle (their performance vanishes at glazing incidence angle). The reason is that the optical path length of the incident light differs at a glazing incidence relative to the norm and the phase difference of reflected wave and incident wave will therefore not cancel each other out (*i.e.*, no complete destructive interference). Moreover, a limited number of materials with low RI find single-layer ARCs for many transparent media (generally low RI substrates) difficult. For instance, for glass (*n* = 1.5), the RI of the coating (according to Equation (1)) should be 1.22. In nature, such a material does not exist and therefore any material with an RI close to this value would have to be used which certainly does not reduce the reflection efficiently. One solution to this problem is using composite single layer ARCs in which the RI of the coating can be tuned by varying the ratio (filling factor) of the components of the composite. This calculation can be made by effective medium approximation such as the Maxwell-Garnet equation [8] (which will not be discussed in detail here; readers are thus referred to [9] and the references therein.):

(3) $$\left(\frac{\varepsilon_{eff}-\varepsilon_{m}}{\varepsilon_{eff}+2\varepsilon_{m}}\right)=f_{i}\left(\frac{\varepsilon_{i}-\varepsilon_{m}}{\varepsilon_{i}+2\varepsilon_{m}}\right)$$

in which $\varepsilon_{eff}$, $\varepsilon_{i}$ and $\varepsilon_{m}$ are the dielectric constant of the effective medium (composite), inclusion and matrix, respectively whereas $f_{i}$ is the filling factor (volue fraction) of the inclusion. Note that the RI of a nonmagnetic medium has the following relationship with the dielectric constant:

(4) $$n=\sqrt{\varepsilon }$$

**Figure 1:**
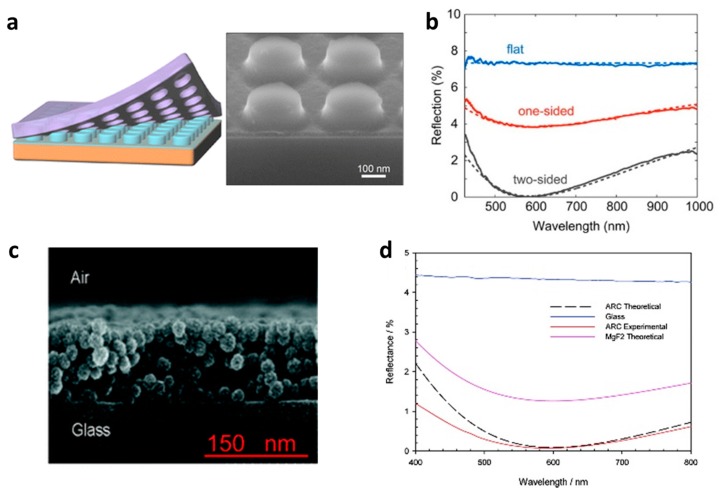
(**a**) Schematic of the one-step nanoimprint lithography fabrication method. The spin-coated sol−gel is patterned by a PDMS stamp to create an effective-index AR coating and SEM image of the sol−gel nanocylinder array on a Si substrate, showing the cylindrical shape of the nanoparticles and the 30 nm residual sol−gel layer (cleaved sample imaged under 52°); (**b**) Measured total reflection spectra for a flat reference (blue), one-side patterned (red), and two-side patterned (black) glass substrates. The dashed lines show the calculated reflection. (Adapted with permission from [10]. Copyright 2015, American Chemical Society); (**c**) Cross-section SEM image of nanoparticle antireflective coating; (**d**) Experimental reflection spectra of glass and ARC and theoretically calculated reflection spectra for ARC and MgF_2_ [12]. (Adapted with permission from [12]. Copyright 2012, American Chemical Society).

Here, the RI of the composite is determined by the mixing of the RI of the components: the higher the volume fraction of the lower RI constituents, the lower the RI of the composite. Accordingly, Polman and co-workers developed a single-layer ARC which is made by sol–gel chemistry and large-area substrate-conformal soft-imprint technology (Figure 1a). The printed pattern is composed of 120 nm tall silica nano-cylinders with a diameter of 245 nm possessing an effective-index (*n* = 1.20) ARC that diminishes the average double-sided reflection of glass from 7.35% to 0.57% (Figure 1b). The RI of the coating indeed can be tuned to realize a broad range of values (1.002 < *n* < 1.44, in theory) which expand the application of the technique for other reflective materials, too [10]. Beside classical uniform single-layer ARCs, many structured or patterned films and surfaces have also been developed which exhibit very low reflection in a range of spectra. These designs include (but are not limited to) silica particles [11,12,13,14,15,16,17,18] (see Figure 1c,d), silica particle-polymer hybrids [19], polymer particles [20] and films [21], composites (silica-Teflon [22], spirooxazine-doped polystyrene [23,24]), vinyltrimethoxy silane films [25], SiO_2_/TiO_2_ particles [26,27], and TiO_2_ film [28] and AlN*_x_* [29]. Although a single-layer (wavelength) ARC is desired in laser and photodiodes [30] and solar cells [31] the narrow band of the reflection dip makes it impractical for many applications such, eyeglasses and displays.

### 2.2. Double-Layer ARC

Double-layer or V-shaped (because of the V-shape of their profile [32,33,34,35]) ARCs are very common in the industry for reducing reflection of a coating from a specific wavelength. In the case of double-layer ARCs, the upper film facing the air usually has the lowest refractive index, and the other layer(s) is (are) made successively based on the ascending order of their refractive indices. In double-layer ARCs, the interference conditions should be fulfilled in order to destructively cancel the bouncing back waves off the surface of the substance. Therefore, the thickness of each single layer is usually quarter or half of the operational wavelength (λ4 and λ2). If the optical thickness of the layers in double-layer geometry obeys the following equations:

(5) $$n_{1}\times d_{1}=n_{2}\times d_{2}$$

the necessary and sufficient index condition in order to reduce reflection down to zero is [33]:

(6) $$n_{1}\times n_{2}=n_{0}\times n_{s}$$

where *n*_0_, *n*_1_, *n*_2_ and *n_s_* are the RI of the air (environment), first (upper) layer, second layer and substrate, respectively and *d*_1_ and *d*_2_ represent the thickness of first and second layers, correspondingly.

**Figure 2:**
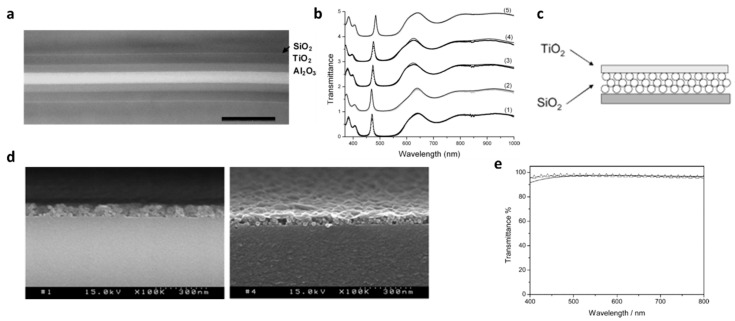
(**a**) TEM micrograph of a TiO_2_ = Al_2_O_3_ bilayer on the Si wafer. The native SiO_2_ layer of ∼1:5 nm can be observed. The scale bar is 200 nm; (**b**) Transmittance spectra of the multilayer coatings for narrow-bandpass filters of the five dichroic filters. The spectra are offset by one for clarity. The dotted curves correspond to the experimental data, and the plain curves correspond to the calculated spectrum. The bandpass wavelength is shifted from 469 to 485 nm (Adapted with permission from [35]. Copyright 2009, The Optical Society); (**c**) Schematic view of double-layered TiO_2_–SiO_2_ with antireflective properties; (**d**) Cross-sectional electron micrographs of (PDDA/SiO2)_6_ and (PDDA/SiO_2_)_6_/(PDDA/nanosheet)_6_ multilayer films after calcination at 500 °C, respectively; (**e**) Calculated (triangles) and measured (dotted line) normal incidence transmission spectra of a TiO_2_–SiO_2_ double-layered film prepared from a (PDDA/SiO_2_)_6_(PDDA/nanosheet)_9_ multilayer assembly. The calculation was based on a 6.5 nm thick TiO_2_/55 nm thick SiO_2_ double-layered structure. (Adapted with permission from [36], Copyright 2006, American Chemical Society).

For instance, a double-layer ARC made of MgF_2_/CeO_2_ or porous silica [34,37,38,39], SiO_2_/TiO_2_ [40,41] or SiN/SiO_2_ [42], MgF_2_/ZnS [30,43,44], MgF_2_/SiNx [45] SiOx/ITO and TiO_2_ = Al_2_O_3_ (Figure 2a,b) [35] would meet the requirements mentioned above and show good ARC performances for silicon. The layers are not necessarily continuous; film made of particles are also considered as a layer such as the one shown in Figure 2c–e. In general, double-layer coatings can be made with various fabrication techniques such as sol−gel/spin-coating [46,47], sol−gel/dip-coating [48,49,50,51], atmospheric pressure chemical vapor deposition [39,52], thermal evaporation [45], plasma-enhanced chemical vapor deposition [43,44,53], reactive evaporation [54], electron beam evaporation [55], and magnetron sputtering [56].

Note that, analogous to a single-layer ARC, each layer of double-layer configuration can be made of composite materials with tunable RI in order to provide more flexibility on the design where the RI of the composite layer are estimated by EM (e.g., Equation (3)). For example, a porous film can be a choice as one or both layers of a double-layer ARC or double layer out of SiO_x_N_y_/porous silicon can reduce the reflectance of silicon down to 0.01% in visible wavelengths [57]. Another example is two stacking layers made of meso and nano-porous silica which substantially enhance the transmission of quartz. Porous silicon combined with diamond-like carbon also reduce the reflectance of silicon in part of the visible spectra [58]. The fragile nature of the porous coating, however, restricts the application of this class of ARC in many applications.

### 2.3. Multilayer Gradient Film as ARC

Deposition of the stack of dielectric layers could also cause destructive interference. This approach has been widely implemented and studied over the last century. The interference effects in the dielectric stacking layers rely on multi-pass light circulation inside the optical cavities formed by the films that render them strongly angle sensitive [59]. According to the Fresnel equation, and based on the Transfer Matrix Method, the reflection of *M* layers (at normal incidence) of slabs with RI equal to ***n*** and the thickness of *l* are calculated by the following equation [60]:

(7) $$R_{i}=\frac{\rho_{i}+R_{i+1}e^{-2jk_{i}l_{i}}}{1+\rho_{i}R_{i+1}e^{-2jk_{i}l_{i}}},\;i=M,\;\text{M}-1,\;\ldots ,\;1$$

in which $k_{i}=\frac{2\pi n_{i}}{\lambda_{0}}$ and $\rho_{i}$ is [60]:

(8) $$\rho_{i}=\frac{n_{i-1}-n_{i}}{n_{i-1}+n_{i}},\;i=1,\;\ldots ,\;\text{M}+1$$

It can thus be inferred that the reflection of a multilayer coating strongly correlates to the thickness and RI of each layer. In principle, finding a proper thickness and RI for multilayers equals optimization of many parameters in order to gain a broadband ARC. This is not simple and needs some optimization and evolutionary algorithms (computer simulation) [61,62]. In fact, according to Schubert *et al.*, “the parameter space generally includes many local minima, which makes deterministic optimization schemes that find the local minima unsuitable” [63]. The interested reader is directed to [61,62] for a detailed description of the related methods.

**Figure 3:**
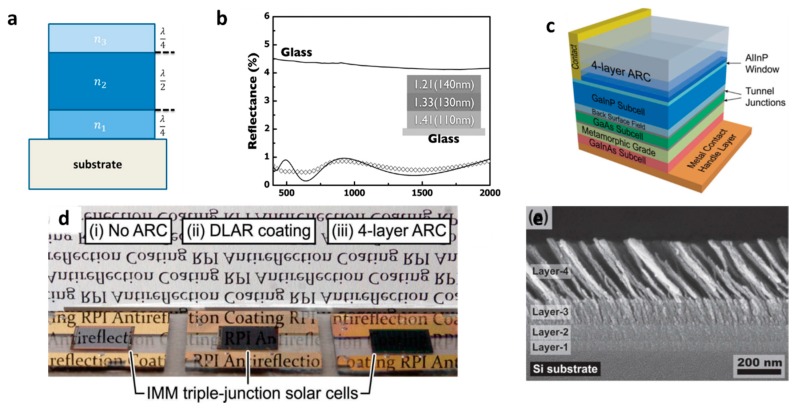
(**a**) Schematic representation of (**a**) multilayer interference coatings (three-layer system as example). (Adapted with permission from [75]. Copyright 2016, American Chemical Society); (**b**) Reflectance curves for (**a**) SMMA69/SMMA46/SMMA30 triple-layered nanoporous film (Adapted with permission from [76]. Copyright 2009, American Chemical Society); (**c**) Schematic layer sequence of an inverted metamorphic (IMM) triple-junction solar cell with four-layer ARC; (**d**) Photograph of three IMM solar cells with (i) no ARC; (ii) DLAR coating; and (iii) four-layer ARC; (**e**) SEM cross-sectional image of the four-layer (TiO_2_/SiO_2_) ARC deposited on a Si substrate [74]. Adapted with permission from [74]. Copyright 2013, John Wiley and Sons).

Multilayer ARCs, similar to double-layer ones, can be a combination of several continuous dielectric films [64,65] or a mixture of porous and continuous layers [66]. Multilayer ARCs can provide multi-dips in reflection spectra at various wavelengths [67] or broadband reflection dips [68]. Loh *et al.* have used multilayers out of porous silica nanoparticle films which resulted in enhancement (reduction) of the transmission (reflection) of glass up to 99% (down to 1%) [69]. The TiO_2_/SiO_2_ multilayer is the most-used combination of dielectric for ARCs. Such a stack shows promising antireflective properties for glass [36,50,70,71,72,73] (see Figure 3a,b) or silicon [74] in visible wavelengths. Such multilayer ARCs are composed of a sequential stack of low and high RI dielectrics with a thickness of quarter-and-half wavelengths as shown in Figure 3c–e.

The adhesion at the interface of the layers, low mechanical stability with higher number of layers and the cost of the process due to the necessity of several deposition runs make the application of such an approach for modern miniaturized optoelectronic devices constrained. Above all, the optimization through experimental trials are very tedious and expensive while the output is very bulky. Accordingly, the quest for an alternative strategy for multilayer ARCs has increased recently, in particular with the emergence of plasmonic and metasurfaces (see plasmonic and metamaterial (metasurface) ARCs). These emerging methods shrink the size (thickness) of the coating considerably because of the light confinement and phase accumulation at a small scale.

### 2.4. Structured Surface as ARC

The surface texturing (structuring or patterning) with a cross-sectional dimension less than that of the incoming light performs as a medium with spatially varying refractive index [77,78]. Contrariwise, a structure with spatial dimensions equal to or larger than the incident electromagnetic wave mostly scatters the light and does not necessarily augment the transmission. According to the literature, Fraunhofer was one of the first users of this concept to realize an ARC when he observed that reflection was decreased upon etching a surface in an atmosphere of sulfur and nitric acid vapors [33]. Almost a century after this pioneering work (observation), a biomimetic method which delivered an anti-glare surface was developed, which is known as the “moth’s eyes” structures [79]. This concept was boosted upon electron microscope analysis of the corneal lenses of moths. Bernhard observed that the outer surface of corneal lenses of moths is covered with an ordered array of conical protuberances, typically of about 200 nm height and spacing [79,80]. Such a bioinspired structure was then realized by Clapham and Hutley [80] (followed by many other researchers in different forms [81,82]) on glass (although it can be realized on any reflective surface) where the coating reduced the reflection of the glass from 5.5% to 0.2%. Although the results were impressive, the developed coating was very delicate and not very applicable [80]. Note that such a bioinspired method is also applied for silicon. Jiang and co-workers used a colloidal silica particle patterned as non-close-packed on silicon as etching mask (for SF6). They showed that such a simple approach can reduce the reflection of silicon down to almost zero in the visible spectrum [83,84].

**Figure 4:**
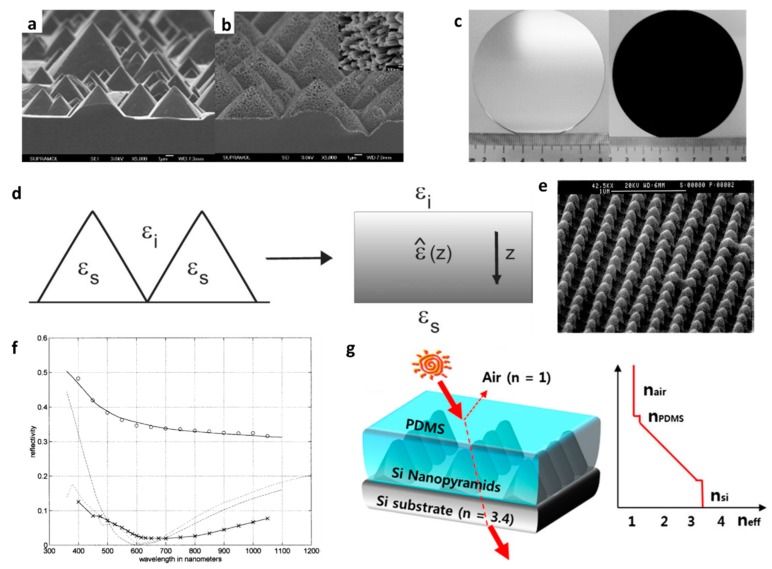
Cross-sectional SEM photographs of (**a**) silicon pyramids created with KOH etching and (**b**) hierarchical structures generated with Ag-assisted etching. Inset: magnified SEM image; (**c**) Photographs of the polished silicon wafer (left) and the hierarchically structured silicon wafer (right) [91]. (Adapted with permission from [91]. Copyright 2009, American Chemical Society); (**d**) In a long wavelength limit, textured surface can be treated as a layer with gradually changing dielectric permittivity tensor ε(z) [89]. (Adapted with permission from [89]. Copyright 2013, The Optical Society); (**e**) Scanning electron micrograph (SEM) photograph of a 260 nm-period 2D grating etched into silicon; (**f**) The upper solid curve and the dashed curve correspond to the reflectivity of a silicon substrate, without coating and with an antireflection quarter-wave, thin-film coating, respectively. The dashed-dot curve corresponds to the reflectivity of a silicon substrate corrugated by an ideal binary 2D SWS surface with a 100 nm thickness and a 0.5 fill factor. These three curves are obtained for normal incidence. The reflectivity measurements include light scattered into a 20° revolution cone around the specular reflection direction. These results are for an angle of incidence of 8° (Copyright IOP Publishing. Reproduced with permission from [92]. All rights reserved); (**g**) Structure and effective refractive index profiles of Si nanostructure deposited via PDMS [93]. (Adapted with permission from [93]. Copyright 2014, Springer).

For solar application, one of the most frequently studied and used approaches is the pyramidal texturing of silicon for ARC purposes [85] (Figure 4a,b). Because of the anisotropic etches of the surface, square-based pyramids are formed on the surface of silicon crystal (wafer) defined by intersecting (111) crystallographic planes [86,87] which can turn the silicon black (Figure 4c). The ARC properties of the texture are strongly germane to the geometry and size of the texture and operating wavelengths. Based on the effective medium approximation, the texture can behave as a gradient index film provided that the incident wavelength (operation range) is larger than the texture size. Conversely, wave rays should be reflected many times until reverted back when the size of the texture is greater than the light wavelength [88]. For the former case, a textured surface can be treated as a film with a gradually varying dielectric permittivity tensor ε(z). The *z* direction is lined up with the pyramid axis (see Figure 4d), with *z* = *0* corresponding to the pyramid tops and *z = d* the pyramid bases. The ε(z) can be calculated by the following equation [89]:

(9) $$\varepsilon_{z}\left(z\right)=f\left(z\right)\varepsilon_{s}+\left(1-f\left(z\right)\right)\varepsilon_{i}$$

in which *ε_s_* and *ε_i_* are the pyramid and substrate permittivity, respectively, while *f(z)* is the filling factor of the pyramid at *z*. Therefore, the shape of the textures and its size dictate the effective properties of the coating and consequently the reflection. However, as outlined above, when the texture size is bigger than the incident wavelength (*i.e.*, shorter wavelengths), the responses of the pattern are not correlated to the incident wavelength but instead are defined only by geometry [88]. However, new trends in thinning the solar cells are to find alternative methods to replace traditional texturing, as the film thickness in thin film solar cells are considerably thinner than the peak-to-valley amplitude of the textured layer [89]. Therefore, large textures are not applicable nor desired for the demand of solar and other optoelectronic industries. However the finer nanostructures such as Moth-eye texture [90] are more likely to find application in photovoltaics industry.

**Figure 5:**
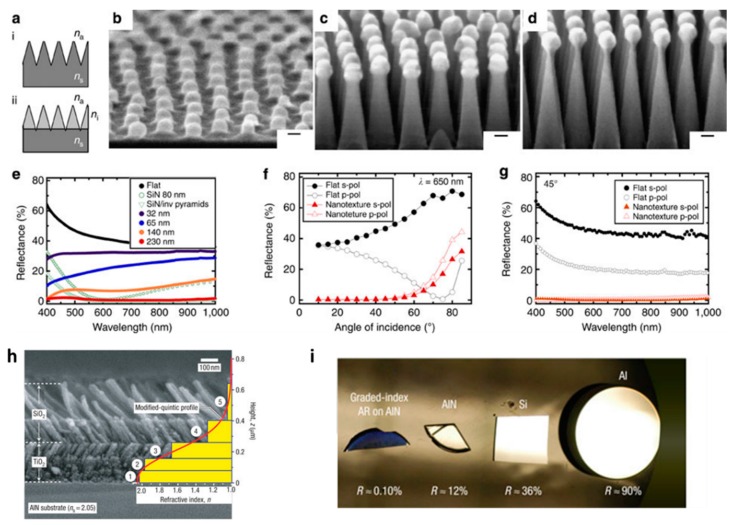
(**a**) Schematic (i) tapered antireflection nanotexture and (ii) tapered nanotexture with graded refractive index; (**b**) 70° Angle cross-sectional scanning electron microscope (SEM) image of close-packed alumina nanostructures formed by infiltrating a 99 kg-mol^−1^ cylindrical phase polystyrene-b-poly(methyl methacrylate) block copolymer. Scale bar, 20 nm; (**c**,**d**) 70° cross-sectional SEM images of nanotextures fabricated by (**c**) 60 s and (**d**) 100 s of plasma etch. Scale bar, 20 nm; (**e**) Measured reflectance *versus* *λ* for a flat Si film, an 80 nm thick silicon nitride film and nanotextures with ℓ = 52 nm and t ranging from 32 to 230 nm. Also shown is the simulated reflectance of an 80 nm thick silicon nitride film on chemically etched inverted pyramids; (**f**) Measured reflectance (at 650 nm) for s- and p-polarization *versus* incidence angle for flat silicon (black circles) and nanotextures with ℓ = 52 nm and *t* = 230 nm (red triangles); (**g**) Measured reflectance of s- and p-polarization *versus* *λ* (45° incidence angle) for nanotextures with ℓ = 52 nm and *t* = 230 nm height (Adapted with permission from [108]; Copyright 2015, Nature Publishing Group); (**h**) Cross-sectional SEM image of graded-index coating with a modified-quintic-index profile. The graded-index coating consists of three TiO_2_ nanorod layers and two SiO_2_ nanorod layers; (**i**) Photograph of a graded-index antireflection coating on AlN and the specular surfaces of AlN, Si and Al (Adapted with permission from [109]. Copyright 2007, Nature Publishing Group).

In general, a porous or discontinuous pattern can provide a gradient layer with better performance than multilayer gradient film. In these cases, the reflection drop is attributed to the effective properties of the coating and not the geometry. The fast-growing advances in nanofabrication methods and state-of-the-art tools boost the design of thin surface with desired ARC properties. Within the last decade, many designs have evolved, leading to the realization of broadband ARCs for silicon and other optoelectronically applicable materials. Steiner and co-workers developed a method which works based on the phase separation of a macromolecular liquid to create nanoporous polymer films. They deposited a polymer blend on the substrate followed by selective etching, leading to generation of a porous polymer film with a very low refractive index [94]. Phase separation in spin-coated polymer film could similarly cause ARC properties as demonstrated by Park *et al.* [95]. Evidently, low RI of the coating reduces the reflection of the substrate and enables an efficient ARC. Lalane *et al.* developed grating etching patterns on silicon by lithography which reduces the surface reflection considerably in visible spectra (Figure 4e,f) [92]. Ha *et al.* [93] made a new scheme where the substrate material is textured and then covered with a polymer film. The developed structure is indeed a (virtual) two-layer ARC whereby the upper film is a low index polymer and the second one is a polymer-silicon rods composite (Figure 4g). Nevertheless, the reflection reduction that they achieved was insignificant. Huang *et al.* [96] developed a texture made of randomly etched nanotips atop a silicon wafer enabling formation of a super broadband ARC (2200 nm bandwidth). The nano-tips were made by a self-masked dry-etching technique where high-density electron cyclotron resonance (ECR) plasma etching was used by reactive gases comprising silane (SiH_4_), methane (CH_4_), hydrogen (H_2_) and argon (Ar) [97]. The low reflection of the texture is attributed to the changes in the refractive index caused by variations in the height of the silicon nanotips (refractive index gradient). Rahman *et al.* used block copolymer self-assembly and plasma etching to produce very ordered surface nanotextures on silicon. The fabricated pattern acts as an effective medium with a graded index refractive index ascending from air to substrate (silicon) thereby diminishing the reflection of the silicon down to zero in visible-NIR wavelengths. The fabrication process, photograph of the sample and corresponding reflection spectra are shown in Figure 5.

Electrochemical etching of silicon is another method used to generate gradient index coating on silicon ultimately applicable for ARC [98]. The porous silicon is formed upon oxidation of silicon in hydrofluoric acid (HF). The electrochemistry cell is composed of platinum as cathode and silicon as anode [99]. Evidently, changing the diameter of the pores and their arrangement (pitch) can influence the filling factor of the pores which is associated with modulation of the refractive index of the silicon. Yet, reproduction (reproducibility) and brittle nature of the porous layer have always been a matter of concern for any application [100]. Indeed, both chemical and electrochemical etching methods have been applied for making a porous structure for ARCs. The etchant (generally acid solution) removes/dissolves certain leachable components at the outer surface of the substance (reflective medium) and leaves or redeposits other constituents, thus forming a skeletonized, porous surface which has a lower refractive index than the substrate [101]. This chemical etching was one of the main methods of choice in the middle of the 20th century [102,103] because of its simplicity and low cost. Creation of porous polymer is an alternative to establish a gradient index ARC [104]. For example, Li *et al.* [105] developed a porous polymer by spin-coating the solution of a polystyrene (PS)-block-poly(methyl methacrylate) (PS-b-PMMA)/PMMA blend onto an octadecyltrichlorosilane (OTS)-modified glass substrate. Thus, a gradient distribution of PMMA domains in the vertical direction of the entire microphase-separated lm is obtained. The process is followed by UV treatment and acetic acid immersion when the PMMA domains are removed leaving a PS porous structure with a gradient RI in vertical direction (normal to the surface). Therefore, they could enhance (reduce) the transmission (reflection) of the glass substrate because of the gradient index condition induced into the coating. This method was later thoroughly examined and improved by Li *et al.* [106]. The trends in polymer-based ARCs can be found in the review by Li *et al.* [107].

Generation of texture or porosity in the medium is not the only method for texturing (gradient index layer formation). The porous or composite coating can be also used for development of coatings with gradient index. For instance, multilayer of TiO_2_ and SiO_2_ graded-index films is deposited by oblique-angle deposition, where the gradient index is built by changing the density of the pores inside the medium (Figure 5h,i) [109]. Chhajed *et al.* [67] also realized a graded index ARC made of nanostructured low-refractive-index silica by oblique-angle deposition. In such a film, the refractive index of silica (SiO_2_) film is reduced from 1.47 to around 1.07. By deposition of such a low index material as the top layer of traditionally known two-layer ARCs, the reflectivity of the silicon is reduced to 5.9% throughout 400–1100 nm in comparison with average reflectivity of 37% for bare silicon [66].

As outlined in the introduction, structuring of silicon by etchant or physical etching methods can turn the silicon black, which is suitable for some photovoltaic application.

## 3. Unconventional ARCs

### 3.1. Absorbing ARCs

In contrast to single-, double- or multilayer ARCs which are made of lossless dielectrics (negligible extinction at operational wavelengths), absorbing layers have also been used for reduction of reflections. There, the reflection reduction not only originates from phase contrast and destructive interference, but the attenuation of the light passing through the absorbing film also contributes to the reduction [110,111,112,113,114,115,116,117,118,119,120].

**Figure 6:**
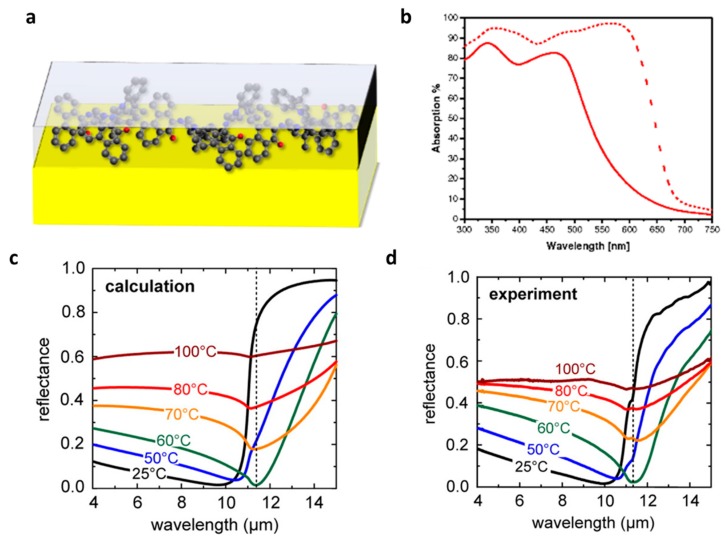
(**a**) Schematic of the geometry used in the metamaterial stack; (**b**) Absorption spectra of PS-SPO composite (solid line) as deposited and upon UV irradiation (dashed line) deposited on 100 nm gold [24]. (Adapted with permission from [24] Copyright 2014, John Wiley and Sons); (**c**) Temperature-dependent reflectance of the checkerboard structure (area coverage *D* = 50%, period = 500 nm, film thickness = 100 nm). At a critical temperature, the reflectance of the sample drops to almost zero for *λ* = 11.3 μm due to an ultrathin film interference interaction between the film and the substrate; (**d**) Calculated reflectance for the sample measured in (**c**) using Fresnel equations and our effective medium approximation. (Adapted with permission from [111] Copyright 2016, American Chemical Society).

Berreman in 1963 analyzed the reflection of LiF on silver film (at 30° incidence angle) and observed a strong sharp absorption band (reflection dip) in infrared wavelength which is attributed to the light coupling to longitudinal optical frequency [112]. However, Oyama and co-workers are among the pioneer researchers who applied the concept of an absorbing layer into the ARC community. They used a glass/TiN_x_O_y_/SiN_x_/SiO_2_ multilayer wherein TiN_x_O_y_ is an absorber for glass. Their optimization showed that the reflectance of the glass can be dimmed down to 0.1 while the transmission is above 70% [113]. Similarly, Kats *et al.* [59] deposited Ge thin film on a gold mirror and demonstrated that the reflection (absorption) of the stack can drop down (goes up) to 20% (80%) at certain wavelengths due to the strong attenuation of the light at the resonance condition. In fact, in a lossy medium, the phase shifts at the interface of the substance and air (in reflection and transmission) are not restricted to 0 or *π* in contrast to lossless dielectrics. Therefore, the phase shifts allow the total phase accumulation (comprising both the interface and propagation phase shifts) to become almost 0 for films much thinner than the conventional quarter-wavelength while resulting in an absorption resonance. Accordingly, the loss of the coating should be high enough to compensate for the phase accumulation when the light passes through ultrathin film [59]. Brongersma and co-workers thoroughly analyzed the system made of a silver mirror covered with a thin layer of Ge. They attributed the high absorption (low reflection) to the coupling of the light to the Brewster mode supported by the structure [114]. Taliercio *et al.* also observed the strong reflection dip in the system of highly doped InAsSb layers lattice-matched onto GaSb substrates which they also attributed to excitation of the Brewster mode [115]. Thin film of silicon on silicon wafer was fabricated by Schlich showing strong reflection dip at various wavelengths [116]. Tischler *et al.* experimentally showed that the 5.1 ± 0.5 nm thick film of J-aggregated dye can critically couple to a single dielectric mirror which reduces the reflection significantly (absorbing more than 97% of incident 591 nm wavelength) [117]. Ding *et al.* [118] and Kumari *et al.* [119] implemented a similar geometry though made of polyvinyl alcohol thin films doped with rhodamine 6 G molecules deposited on optically thick silver substrates illustrating nearly unity absorption (zero reflection) at 550 nm. We, the authors of present review, have also recently demonstrated that a hybrid film made of spirooxazine (SPO) in a polystyrene (PS) matrix can perform as a switchable reflector/antireflector (reflector/absorber) coating although the thickness of the layer is far below the quarter-wavelength [24,120]. The geometry is shown schematically in Figure 6a,b where a 50 nm PS-SPO hybrid film is spin-coated [23] on an optically thick gold film. The UV irradiation of the coating triggered the dye molecules and the coating turned to an absorbing (lossy) medium. Accordingly, the reflection of the gold film which is around 95% at 600 nm drops to almost zero when the SPO are activated through UV illumination. Kats *et al.* used VO_2_ thin film on sapphire substrate for creation of a tunable reflector (absorber). They demonstrated that heating of the film above transition temperature can change the reflectivity from a few percent up to 80% at 11 µm wavelength [110]. In other words, reflection manipulation by temperature (heating/cooling) is possible by using phase-changeable materials. Such a phase-changing performance does not limit the application of VO_2_ to visible spectra but can also be realized in IR wavelengths [111] (Figure 6c,d). Thin silicon film deposited on an aluminum mirror was analyzed by Mirshafieyan and Guo where they observed a strong reflection drop depending on the thickness of the coating. They attributed the observation to critical coupling of light to the second-order resonance mode of the optical cavity made of a thin silicon film on aluminum surface [121].

Although some part of the light is lost due to dissipation through the absorbing layers, there are numerous circumstances wherein high transmission is not as vital as bright light-emitting displays represented by a cathode ray tube [113], bolometers and stealth technology [59]. Hence, absorbing ARCs can open up an entirely new area of research in optics and photonics. Nevertheless, this class of coatings is not applicable to many optical products because of the large attenuation and loss (transmission loss).

### 3.2. Plasmonic and Metamaterial (Metasurface) ARCs

**Figure 7:**
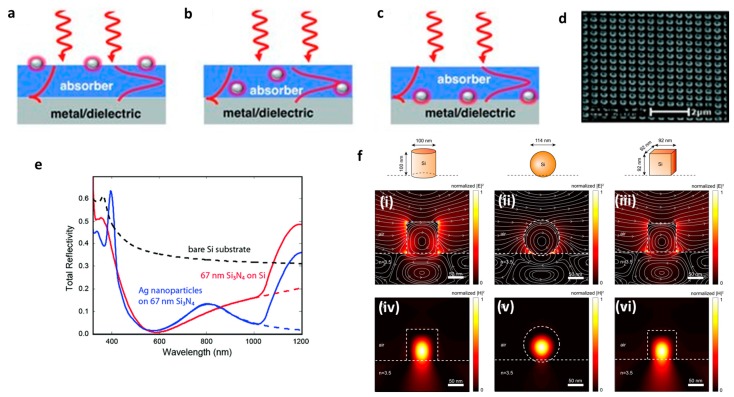
Generalized scatterers for coupling into waveguide modes in a solar cell. Scatterers can consist of particles on the top (**a**); middle (**b**); or back (**c**) of the solar cell and could contain layers of metal, dielectrics, transparent conducting oxides, or air on the back surface. Incident sunlight is then scattered into photonic or SPP modes depending on the scattering object and incident wavelength of light (Adapted with permission from [134] Copyright 2010, John Wiley and Sons); (**d**) The SEM image of a silver nanoparticle array, fabricated by EBL; (**e**) Measured total reflection spectrum from a 300 μm crystalline Si cell coated with 67 nm Si_3_N_4_ (red) and the same sample with an optimized Ag particle array on top (blue). The particles reduce the reflection for wavelengths above 800 nm, improving the incoupling of light into the Si substrate. The dashed lines are extrapolated data representing the reflection from a semi-infinite substrate. The calculated reflectance of a semi-infinite Si substrate is shown for reference (dashed black line) (adapted with permission from [129]; Copyright 2011, American Chemical Society); (**f**) (i–iii) Vertical crosscuts through the center of all three particles in the plane parallel to the E-field of the source, showing the normalized |E|^2^ (color) and electric field lines (gray). The particle surroundings and air-substrate interface are indicated with white dashed lines. The respective geometries are shown above the figures. The displacement current loops are clearly visible (iv–vi). The same crosscuts as in (i–iii), now showing the normalized |H|^2^ (color) of the MD modes. The magnetic field lines are not plotted since H∼0 in this plane (perpendicular to source H⃗) (adapted with permission from [135]; Copyright 2013, The Optical Society).

As emerging fields of optics and photonics, plasmonics and metamaterials (metasurfaces) have also been used for ARCs; metallic nanoparticles or nanostructures were fabricated as an upper layer in order to couple the light to the waveguide mode of the substrate or scatter preferentially the light toward the substrate. In principle, because of the forward scattering of the incident light by the plasmonic nanostructures or particles (Figure 7a–c) [122,123,124,125] or electromagnetic confinement around the top particles [126,127,128], the reflectivity is significantly reduced while providing nearly perfect impedance matching of light to the substrate [129]. Note that the plasmonic ARCs possess one distinctive factor which distinguishes them from their dielectric-based counterpart: partial absorption of the light. This intrinsic loss restricts the applicability of the plasmonic solar cells to devices wherein the localization and confinement of the light is an advantage, including thermal photovoltaics, thermal collectors and absorbers. In other words, the reflection reduction occurs at the expense of the partial parasitic ohmic loss in the metal (analogous to absorbing ARCs), and can therefore not be used in some optic devices (e.g., glasses, telescopes, lenses, *etc.*).

Polman and co-workers [129] developed a plasmonic ARC made of silver nanostructures on Si_3_N_4_ layers for silicon wafer. The reflection is dropped throughout the visible spectrum which was attributed to a combination effect of plasmon scattering toward the substrate as well as interference through the dielectric film (Figure 7d,e). Aluminum can be a candidate for similar geometry (instead of silver). Theoretical calculation of Zhang *et al.* showed that the performance of aluminum-based ARCs can be superior to that of their silver and gold counterparts because of the broadband nature of absorption in aluminum [130]. The great potential of aluminum was further experimentally proven by Villesen *et al.* [131], and Maier *et al*. [132] where they showed that aluminum-based coating can even enhance the external efficiency of GaAs thin film photodiode since the far-field scattering effects of aluminum take over parasitic absorption in visible spectra (where it is of paramount interest in solar collection) [132].

Note that the details of fabrication methods and procedures are discussed in a review by Cai and Qi [133] and will not be repeated here.

Since the scattering is not an exclusive property of metals, the nanostructures can be made of a non-metallic component in order to provide the forward scattering toward the substrate. The resonance of non-metallic clusters could similarly show very promising ARC performance as demonstrated by Spinelli *et al.* [136]. They demonstrated that a regular array of low aspect-ratio silicon nano-cylinders etched into a silicon wafer displays an average reflectivity as low as 1.3% in visible and NIR wavelengths. In such a geometry, the resonant Mie modes of the patterns (scatterers) intensely interact with the incident wave. Their coupling to the substrate results in a strong preferential forward scattering due to the high-mode density in the high-index substrate, thereby causing the reflection to vanish (Figure 7f). Although the substrate surface is only covered with a 30% array of scatterers, the interaction of the features with incoming light is very large. This is because of the resonant nature of the process which results in a large cross-section [136]. The shape and size of the particles influence the coupling efficiency and performance of the coating. Figure 7f shows the role of the shape on the field confinement and coupling into the substrate where it is found that cylinders outperform other shapes [135]. A silicon (solar cells) antireflective coating, *i.e.*, arrays of TiO_2_ nano-cylinders deposited on a Al_2_O_3_ passivated silicon developed by Spinneli *et al.*, shows a broadband ARC covering 420–980 nm wavelengths. Strong forward scattering of the Mie resonances of the TiO_2_ NCs is the reason behind the reflection-reducing characteristics of the coating [137].

The scattering of particles near a surface is studied thoroughly both theoretically and numerically in [138] and will not be discussed here as it is far beyond the scope of this review.

In addition to those already outlined, different plasmonic and metamaterial-based ARCs have been developed, including semiconductor substrates such as gold-SiN*_x_* [139], silver-silica composite [140] and indium-TiO_2_ patterns [141].

**Figure 8:**
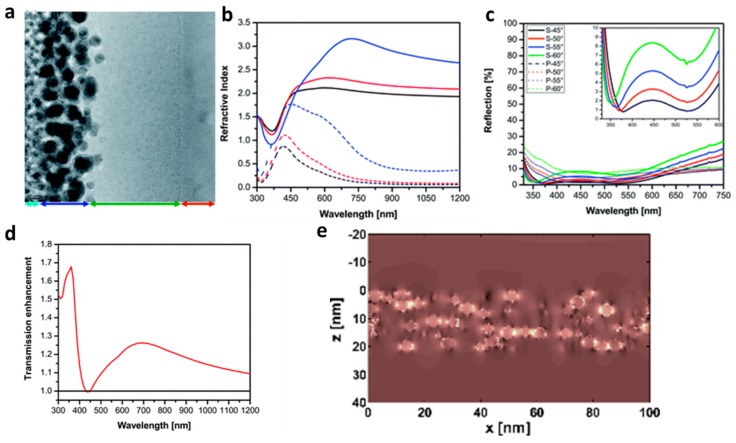
(**a**) Cross-sectional image of plasmonic nanocomposite in which the arrows indicate each of the samples. Light blue, blue, green and red arrows represent the platinum (top adhesion layer for cutting of the sample), nanocomposite, SiO_2_ layer and silicon wafer, respectively; (**b**) Complex refractive index of the Ag–SiO_2_ nanocomposite with 15% (black), 20% (red) and 40% (blue) filling factors. Solid and dotted lines represent the real and imaginary parts of RI, correspondingly; (**c**) Reflection spectra of 20 nm Ag–SiO_2_ with 20% (red) and 30% (blue) filling factors deposited atop 50 nm SiO_2_-coated silicon measured at different angles of incidence with s—(solid lines) and p—polarization (dotted lines); (**d**) Calculated transmission enhancement of silicon by plasmonic coating, which is calculated by normalization of the transmission of coated silicon in comparison to the bare one. The black line shows the normalized transmission of bare silicon while the red curve is the normalized transmission of the Ag–SiO_2_ nanocomposite deposited on SiO_2_-coated silicon (adapted with permission from [142]; Copyright 2014, Royal Society of Chemistry); (**e**) Amplitude of the electric field in a cross-section through the plasmonic nanocomposite (between *z* = 0 nm and *z* = 20 nm). The incident’s linearly polarized plane wave propagates in the +*z*-direction. The plasmonic nanocomposite has a filling fraction of 20% and consists of randomly arranged non-touching silver nanospheres with a diameter of 4.1 nm embedded in a generic mondisperse dielectric material (ϵ = 2.25). The amplitude is shown at an incident wavelength of 430 nm. (Adapted with permission from [143]. Copyright 2014, MDPI).

Aperiodic patterns or dispersion of random shapes and size scatterers or resonators can further broaden the reflection dip because of the overlap of the resonances of resonators with different shapes and sizes. We, the authors of this manuscript, have accordingly used this concept and developed an ultrathin ARC for silicon using a nanocomposite made of silver nanoparticles enclosed in a silica matrix [142] or gold-silica nanocomposite [143] deposited on a silica-coated silicon wafer (Figure 8a). The strong dispersivity of the plasmonic nanocomposite enables realization of two virtual geometries in one design: gradient Rayleigh and reverse-Rayleigh geometries. In other words, above the resonance, the top layer RI is smaller than the second layer (conventional gradient Rayleigh ARC) while below the resonance, the RI of the outer layer is greater (reverse-Rayleigh geometry) (Figure 8b). Such a hybrid coating which is almost angle insensitive (Figure 8c) because of its small thickness, enables reflection reduction across the visible frequency and turns the reflective silicon wafer to a black surface. In comparison to other plasmonic ARCs, ohmic loss of the metallic components reduce the optical transmission into the substrate; nevertheless, the loss is not significant in comparison to the gained transmission enhancement (Figure 8d). FDTD analysis revealed the strong confinement of the field amid nanoparticles in resonance frequency [143] (Figure 8e). The fabrication method of the aforementioned nanocomposites was co-sputtering; however, other methods such as thermal dewetting [144] (annealing) are also used for similar purposes. Many aperiodic systems are studied in the literature such as gold/silver clusters [145], gold-TiO_2_ composite [146], gold-silica [147], silver-SiN*_x_* [148] and Al-SiN*_x_* [149]. However, photocurrent enhancement caused by Al nanoparticles sited atop a silicon diode are compared in periodic and aperiodic arrangement by Uhrenfeldt *et al.* [150]. They found in an experimental work that a periodic geometry is superior to an aperiodic one due to an additional Fano-like resonance, which boosts the photocurrent augmentation of the periodic array when compared with the random geometry [150]. Although research of these novel metamaterials/plasmonic methods is still in its infancy, their miniaturized structure and low material cost, in addition to their thinness make them an outstanding candidate for future industrial use.

## 4. Applications

As outlined in the introduction, there are many applications for antireflective coatings; they are not only for glasses [151], lenses [152] and astronomical purposes [153], but state-of-the-art optoelectronic devices are also highly dependent on ARCs. Light-emitting diodes (LEDs) [154], solar cells [155] (including thin-film ones [156]), thermophotovoltaics [157], lasers [158], displays [32] and photolithography [159], are among the main emerging applications of ARCs.

Despite the broad range of applications for the aforementioned conventional ARCs, the real application of unconventional ARCs is still in the theoretical stage. In fact, the optical losses through the metallic component—the most essential component of the unconventional ARCs—remains a challenge for real-world application. Specifically, the metallic components (e.g., gold and silver) are materials undesired in the silicon industry and efforts have therefore been made to develop novel ARCs without metallic components. Metal nitrides are the most recently proposed alternative and show promising optical performances without being lossy [160,161]. Nevertheless, as the concept of plasmonic antireflective coating is rather new, a great deal of effort still needs to be made in order to shift the concept from the laboratory to real-world devices.

## 5. Summary

Conventional single, double or multilayer antireflective coatings (ARCs) are constrained by thickness (quarter/half-wavelength) requirements for interferences. This fundamental limitation delays further improvement of their performance, particularly as a result of the current demand for thinness and broadband application in many optoelectronic devices.

Ever-growing progress in both nanofabrication and nanophotonics have matured the field of ARCs, and state-of-the-art ARCs are ultrathin and broadband and iridescent free. These new designs and approaches could boost the performance of many devices for which light reflection is unwanted, such as solar cells and LEDs. However, the long-term stability, mechanical robustness and cost of the fabrication of plasmonic- and metasurface-based ARCs remain obstacles to driving the concept from the laboratory scale to real application.
